# Safe and highly efficient adaptation of potentially explosive azide chemistry involved in the synthesis of Tamiflu using continuous-flow technology

**DOI:** 10.3762/bjoc.15.251

**Published:** 2019-10-30

**Authors:** Cloudius R Sagandira, Paul Watts

**Affiliations:** 1 Nelson Mandela University, University Way, Port Elizabeth, 6031, South Africa

**Keywords:** azide chemistry, continuous flow synthesis, hazardous, safe, Tamiflu

## Abstract

Tamiflu is one of the most effective anti-influenza drugs, which is currently manufactured by Hoffmann-La Roche from shikimic acid. Owing to its importance, more than 60 synthetic routes have been developed to date, however, most of the synthetic routes utilise the potentially hazardous azide chemistry making them not green, thus not amenable to easy scale up. Consequently, this study exclusively demonstrated safe and efficient handling of potentially explosive azide chemistry involved in a proposed Tamiflu route by taking advantage of the continuous-flow technology. The azide intermediates were safely synthesised in full conversions and >89% isolated yields.

## Introduction

**Scheme 1:**
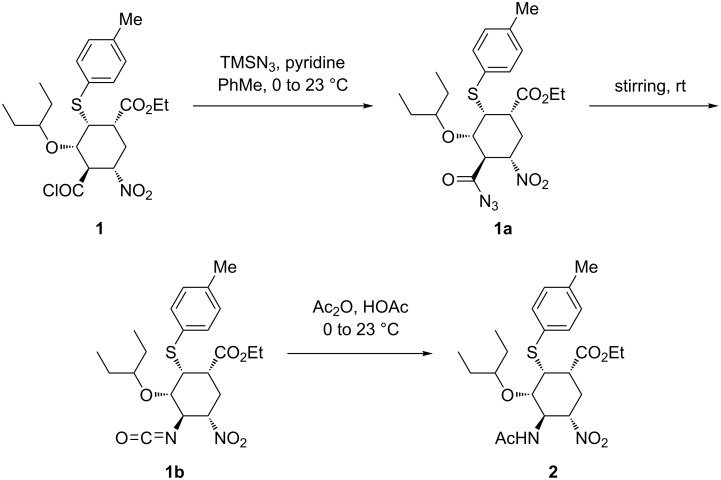
Handling of azide chemistry in Tamiflu synthesis by Hayashi and co-workers [14].

**Figure 1:**
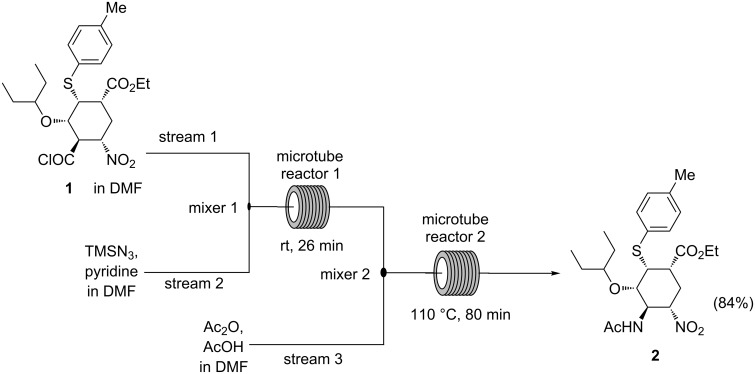
Synthesis of compound **2** from acyl chloride **1** via Curtius rearrangement using a continuous-flow system [14].

Tamiflu is currently one of the most important drugs available to combat the influenza virus and this has seen immense research efforts by the scientific community to exclusively focus on the development of new, better and practical approaches to manufacture this drug [1–2]. More than 60 synthetic routes have been developed towards Tamiflu to date [1–3]. However, most of these synthetic approaches suffer from the use of potentially hazardous azide chemistry, thus raising safety concerns [4] and eventually ruled out for large scale synthesis in batch systems [1–2]. The importance and use of azide chemistry in organic chemistry synthesis is well documented [5]. However, there are potential hazards associated with its application thus posing safety concerns [6–8]. From the viewpoint of azide chemistry, the synthesis of Tamiflu is very interesting in many aspects, because azide chemistry is extensively utilised. Most reported synthetic routes towards Tamiflu employ the potentially explosive azide chemistry to introduce *N*-based substituents on the drug [5–8]. Furthermore, the current industrial production route by Hoffmann-La Roche is no exception. Hoffmann-La Roche worked closely with a firm that specialises in azide chemistry to develop its industrial process [1–2,6,9]. Numerous reported routes demonstrate amazing potential and ingenuity in the Tamiflu molecule assembly. However, most of them are not amenable to easy scale-up due to the safety concerns associated with azide chemistry [1–2]. Therefore, the development of alternative practical and safe processes for Tamiflu synthesis, which can be adapted at large scale is imperative. The safety concerns associated with the use of azide chemistry prompted the chemical community to develop azide-free synthetic routes [1–2,7,10–11]. However, routes involving azide chemistry proved to be more superior in most instances than azide free alternatives [2,12]. Hayashi’s group developed highly efficient two ‘one-pot’ sequences towards Tamiflu at gram-scale, which proceeded in 10 steps with an outstanding 60% overall yield [1,13]. The approach required five isolations only. Unlike Magano [1], the authors could not avoid the use of the potentially explosive azide chemistry. The azide intermediate was not isolated to address the safety concerns posed by azides. Positively, their approach was characterised by low catalyst loading, no protecting group chemistry and absence of halogenated solvents [1,13]. Generally, this approach is attractive for large scale manufacturing, however, the safety concerns posed by the use of azide chemistry needs to be addressed especially at large scale where the risk is very high. In an effort to address the safety concerns raised by the potentially explosive acyl azide **1a** (Scheme 1), Hayashi and co-workers [14] demonstrated the handling of the Curtius rearrangement reaction (transformation from acyl azide **1a** to isocyanate **1b**) in a microreactor system (Scheme 1 and Figure 1). Acyl azide **1a** is a potentially explosive compound because of its nitro and azide moieties [14] and its safety concerns need to be dealt with for large scale synthesis. Safety in this reaction was achieved by in situ formation and consumption in flow of the hazardous intermediates (azide **1a** and isocyanate **1b**) (Scheme 1 and Figure 1).

Continuous-flow synthesis offers the generation and consumption of dangerous intermediates in situ preventing their accumulation, thus it represents a potential solution for dealing with hazardous reaction intermediates and products [15]. Additionally, microreactors can handle exothermic reactions extremely well, due to the inherent high surface area to volume ratio and rapid heat dissipation [16] unlike the conventional batch process. Continuous-flow production may certainly enhance the green metrics of synthesis in several ways [17] and their work clearly demonstrated the possibility of using continuous-flow systems as a way of solving the problems associated with handling hazardous intermediates and products in the synthesis of Tamiflu.

**Scheme 2:**
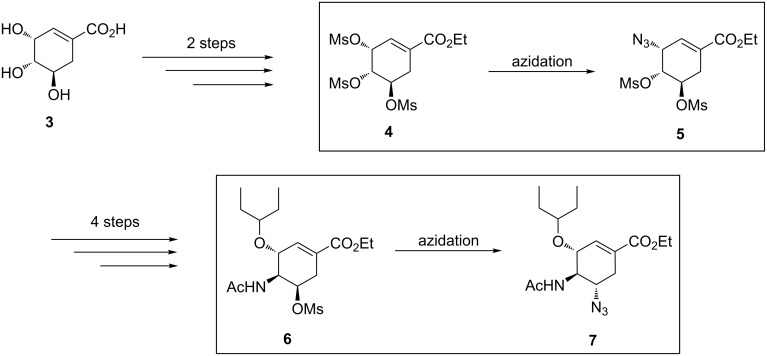
Azide chemistry in the synthesis of Tamiflu.

With this in mind, we investigated safe ways of handling this important but hazardous azide chemistry in Tamiflu synthesis by using continuous-flow technology. The two steps involving azide chemistry in flow are reported herein (Scheme 2), with the vision of further integrating the other steps towards continuous-flow total synthesis of this drug. To this effect, we have already reported continuous flow shikimic acid (**3**) esterification; the first step in the synthesis of Tamiflu [18].

## Results and Discussion

**Scheme 3:**
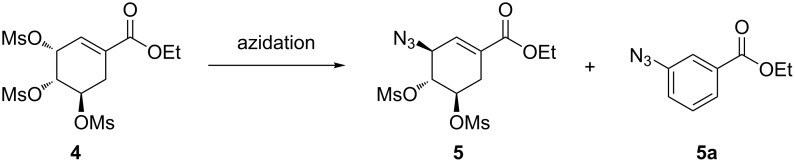
Azidation of mesyl shikimate **5**.

**Continuous-flow synthesis of ethyl (3*S*,4*R*,5*R*)-3-azido-4,5-bis(methanesulfonyloxy)cycohex-1-enecarboxylate (5).** Mesyl shikimate azidation is a pivotal step in our proposed Tamiflu route. Mesyl shikimate **4** in the presence of a suitable azidating agent undergoes a highly regio- and stereoselective nucleophilic substitution of allylic *O*-mesylate at the C-3 position affording azide compound **5** (Scheme 3).

Nie and co-workers [19] reported the treatment of mesyl shikimate **4** with NaN_3_ (4 equiv) in aqueous acetone (Me_2_CO/H_2_O 5:1) at 0 °C for 4 h in batch to afford azide **5** in 92% yield. The use of high temperatures was detrimental to azide **5** yield due to the formation of the side product ethyl 3-azidobenzoate (**5a**). For example, at room temperature for 3 h or at 50 °C for 2 h, the aromatic side product **5a** was obtained in 16% and 81% yield, respectively. Consequently, the reaction was strictly done at 0 °C and below. Therefore, the use of NaN_3_ in high equivalence (4 equiv) was to probably improve reaction kinetics at low temperatures. Karpf and Trussardi [9] reported the synthesis of azide **5** from mesyl shikimate **4** and NaN_3_ (1.1 equiv) in DMSO for 3 h at room temperature in batch. Unlike Nie et al. [19], they used only a slightly excess of NaN_3_ (1.1 equiv) at room temperature to achieve good yields, however, the use of DMSO as solvent made the product isolation more difficult. Despite also acknowledging aromatisation side reactions, this communication did not report actual figures on yields for both desired azide **5** and side product **5a**. Kalashnikov et al. [20] observed that the slightly basic nature of NaN_3_ also contributed to side reactions. To improve on Karpf and Trussardi’s approach [9], they utilised less basic NH_4_N_3_ (1.5 equiv) generated in situ from NH_4_Cl from NaN_3_ in MeOH to azidate mesyl shikimate **4** for 5 h at room temperature in batch to afford desired azide **5** (95%) and side product **5a** was not quantified [20]. Ethyl 3-azidobenzoate (**5a**) is the common reported side product [19–20] for the mesyl shikimate **4** azidation reaction (Scheme 3).

We herein present a comprehensive study on various mesyl shikimate **4** azidation procedures; with goal of safely and selectively making azide **5** in flow.

**Figure 2:**
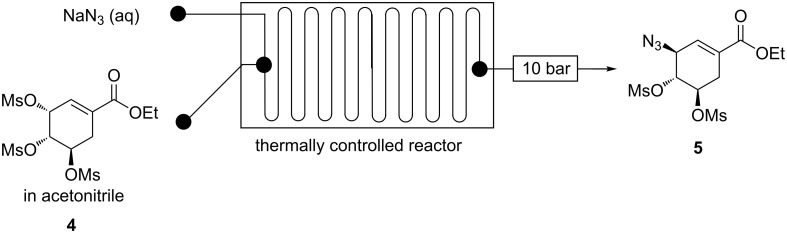
Continuous-flow system for C-3 azidation of mesyl shikimate using aqueous sodium azide.

**Continuous flow C-3 mesyl shikimate azidation using sodium azide (NaN_3_)**. A continuous flow system fitted with a 19 µL reactor (Chemtrix) was used to optimise the synthesis of azide compound **5** from mesyl shikimate **4** using aqueous NaN_3_ (Figure 2). Initial studies had shown the same conversions in both acetone and acetonitrile as solvents. Although acetone is a greener solvent than acetonitrile [21–22], its use was accompanied with eventual microreactor blockage caused by a resulting precipitate from the acetone/aqueous NaN_3_ mixture. Fortunately, acetonitrile is also an acceptable green solvent [21–22]. Furthermore, acetonitrile has a higher boiling point than acetone which is desirable for high temperature reaction interrogation. Consequently, acetonitrile was the preferred solvent for mesyl shikimate **4** for further optimisation in continuous-flow systems.

**Figure 3:**
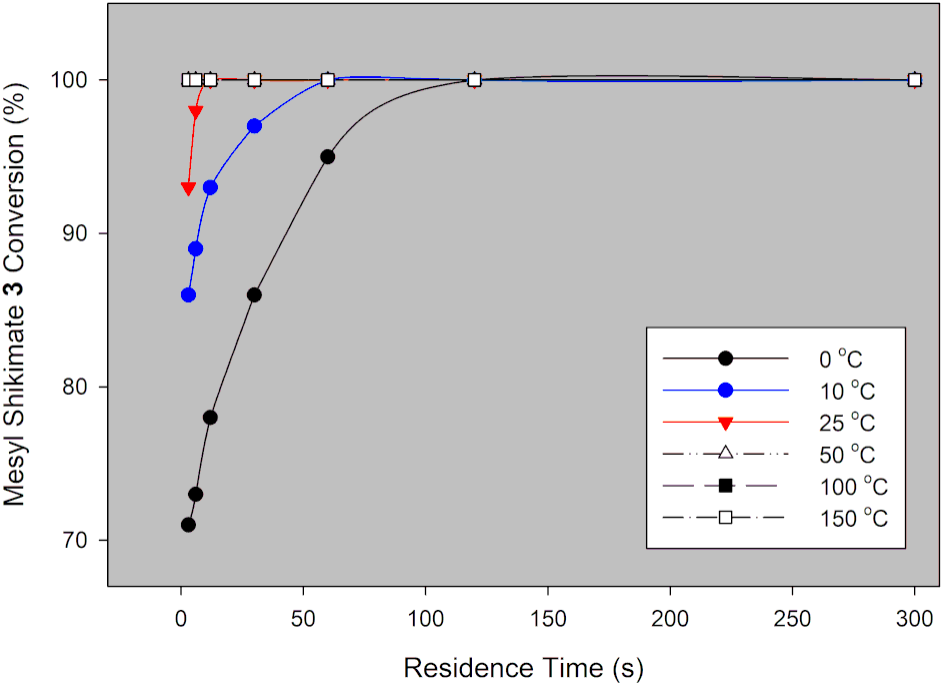
Mesyl shikimate azidation conversion in a continuous-flow system using NaN_3_.

**Figure 4:**
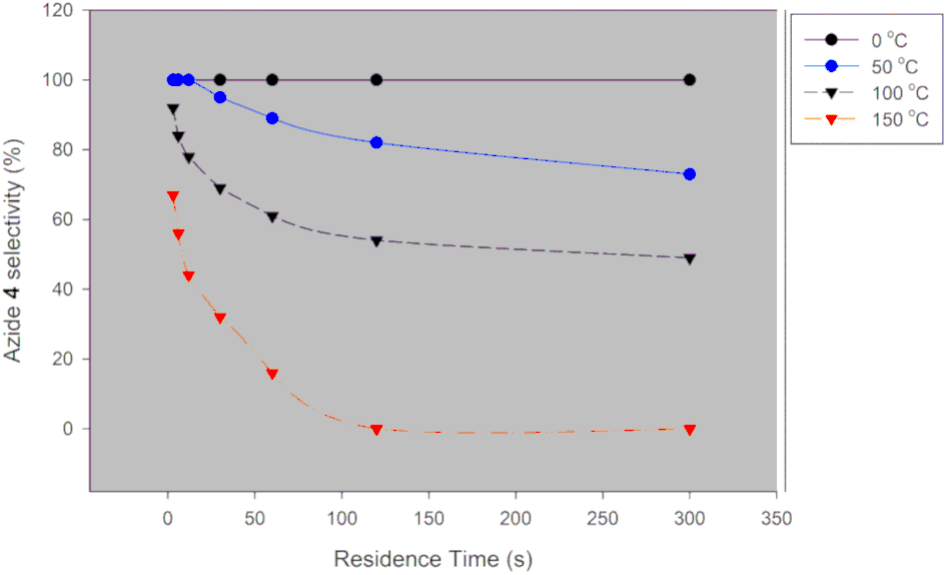
Desired azide **5** selectivity in a continuous-flow system using NaN_3_.

Mesyl shikimate **4** (0.1 M) in acetonitrile was treated with aqueous NaN_3_ (0.11 M, 1.1 equiv) in a thermally controlled microreactor system (Figure 2). Generally, good mesyl shikimate conversions were obtained. As aforementioned, the reaction affords two products, the desired azide compound **5** and the side product **5a** (Scheme 3). The findings on the effect of various reaction conditions on conversion and selectivity are presented graphically (Figure 3 and Figure 4).

It is evident that mesyl shikimate conversion increases with increase in residence time and temperature (Figure 3). At 50 °C and above, full conversions were achieved at incredibly low residence times. Full conversion was achieved at 50 °C, 3 s residence time and 71% conversion at 0 ° C and 3 s residence time (Figure 3). Product selectivity to azide **5** is shown in Figure 4.

**Figure 5:**
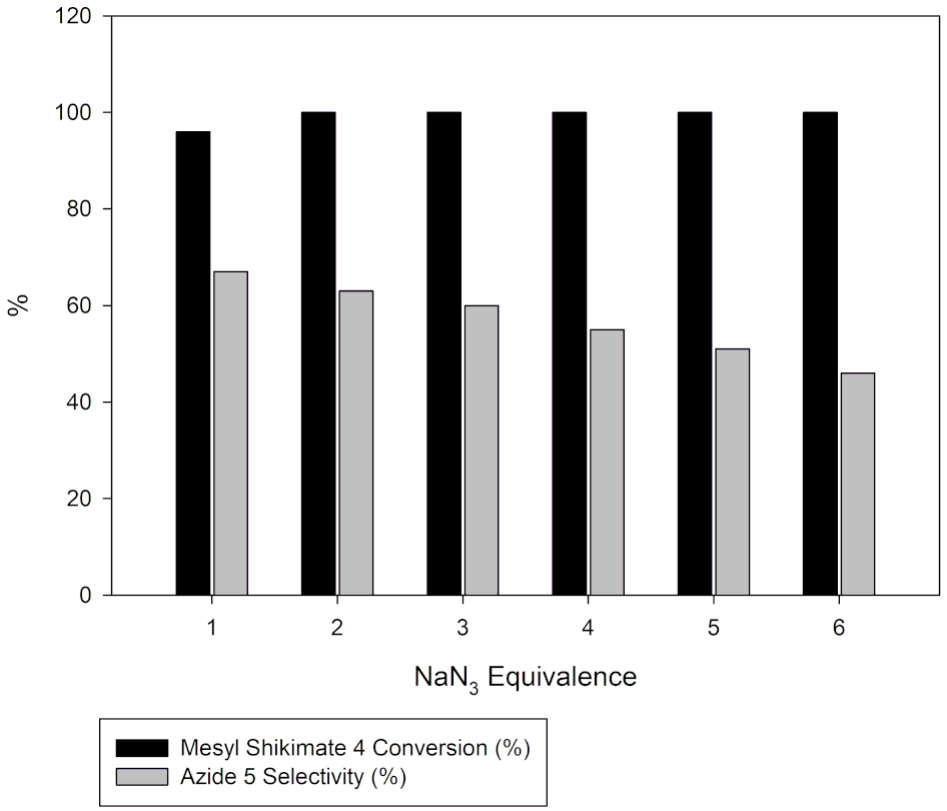
Effect of NaN_3_ concentration on mesyl shikimate **4** conversion and azide **5** selectivity.

Generally, selectivity decreases with increase in residence time and temperature (Figure 4). However, there is 100% selectivity towards the desired azide **5** at 0 °C at all the investigated residence times with conversion ranging from 71% to 100%. There was full conversion at 150 °C with 67% and 0% azide **5** selectivity observed at 3 s and 300 s, respectively. At 50 °C, full conversion was achieved with 100% and 73% azide **5** selectivity at 3 s and 300 s, respectively (Figure 3 and Figure 4). It is evident that high temperatures favour the undesired aromatic azide compound **5a**. Nie et al. [19] obtained 16% yield of the aromatic compound **5a** at room temperature for 3 h and 81% at 50 °C for 2 h in batch. The undesired aromatic azide **5a** forms from the desired azide product **5** via elimination and aromatisation [19–20]. Kalashnikov and co-workers [20] further ascertained that the considerable basicity of NaN_3_ caused the side reaction. Therefore, we investigated the effect of NaN_3_ concentration on the reaction. Figure 5 illustrates the effect of NaN_3_ molar equivalent on mesyl shikimate **4** conversion and selectivity of the desired azide **5** at 150 °C and 12 s residence time.

The selectivity towards azide **5** decreases with increase in NaN_3_ concentration (Figure 5). Contrary, mesyl shikimate **4** conversion is improved with increased NaN_3_ amounts (1 and 2 equivalents). The unexpected decrease in selectivity with increase in NaN_3_ concentration can be understood by considering Kalashnikov and co-workers’ [20] explanation on NaN_3_ basicity-induced aromatisation resulting in undesired azide **5a**. Therefore, an excess of NaN_3_ increases reaction basicity resulting in the undesired azide **5a** being favoured.

**Figure 6:**
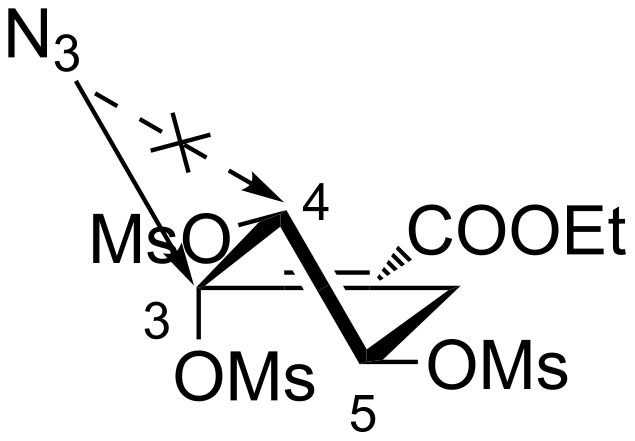
Regio- and stereospecific nucleophilic -N_3_ group attack.

The continuous flow mesyl shikimate **4** azidation was highly regio- and stereoselective to the C-3 position [9,19–20,23]. The two OMs groups at C-4 and C-5 remaining intact as reported in batch [9,19–20]. The highly selective C-3 azidation is reasonable and easily understood because the C-3 position is much more reactive (allylic position) and less hindered (Figure 6).

The optimum conditions in flow for this reaction were found to be 1.1 equivalents of NaN_3_, 50 °C and 12 s residence time affording full conversion towards the desired azide **5**. Despite of high temperatures, long reaction times and basicity being detrimental to the selectivity of the desired azide **5** in batch, it is evident from our study that the use of a microreactor significantly improved the selectivity and massively reduced the reaction times. The production of azide **5** in 100% conversion simplified the purification procedure. Most importantly, microreactors improved safety as the potentially explosive azide chemistry was safely investigated even at very high temperatures thus making the process greener.

Although we successfully developed a safe and efficient continuous-flow procedure for the synthesis of azide **5** from mesyl shikimate **4** by using NaN_3_ (aq), the vision, however, is further integrating all the steps in Tamiflu synthesis. We knew that the current step will not be compatible with the subsequent step when considering telescoping as it requires anhydrous conditions. This prompted us to investigate whether alternative azidating agents, which are soluble in organic solvents, such as diphenyl phosphoryl azide (DPPA), trimethylsilyl azide (TMSA) and tetrabutylammonium azide (TBAA) are suitable for the reaction.

**Figure 7:**
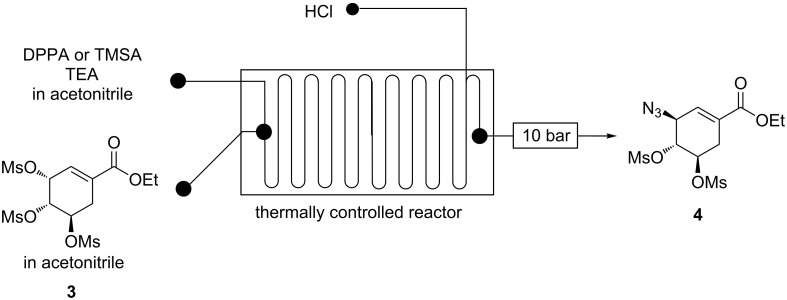
Continuous-flow system for C-3 azidation of mesyl shikimate using DPPA or TMSA.

**Continuous flow C-3 mesyl shikimate azidation using either DPPA or TMSA.** The use of either DPPA or TMSA as the azidating agent for mesyl shikimate **4** was investigated in a Chemtrix continuous-flow system (Figure 7).

Mesyl shikimate (0.1 M) was treated with a mixture of either DPPA or TMSA (0.11 M, 1.1 equiv) and TEA (0.12 M, 1.2 equiv) in a continuous-flow system (Figure 7). The reaction was quenched with aqueous HCl (0.05 M, 0.5 equiv) within the flow system.

**Figure 8:**
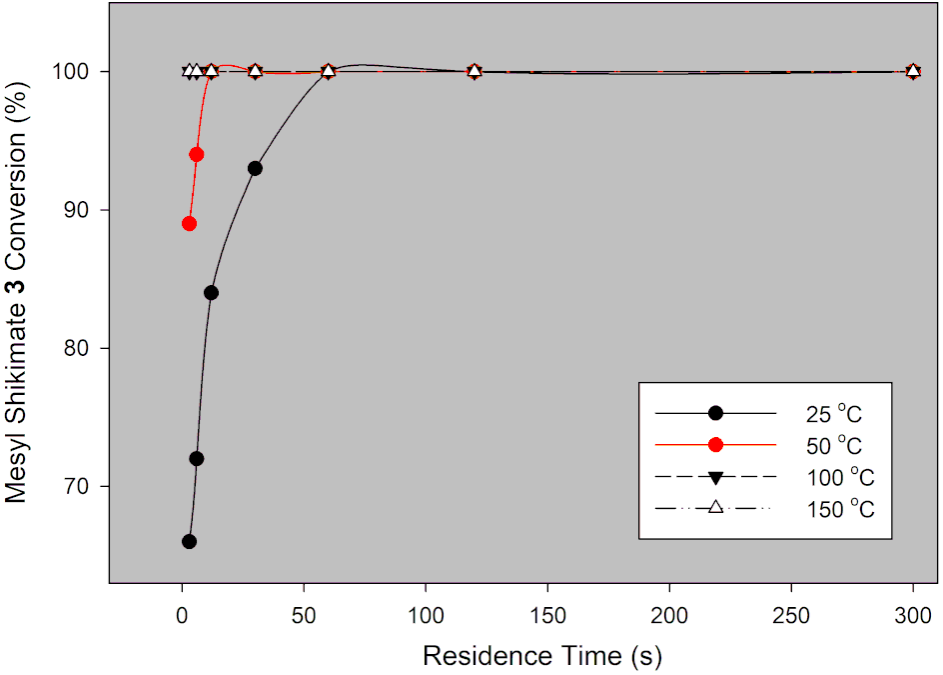
Mesyl shikimate azidation conversion in a continuous-flow system using DPPA.

**Figure 9:**
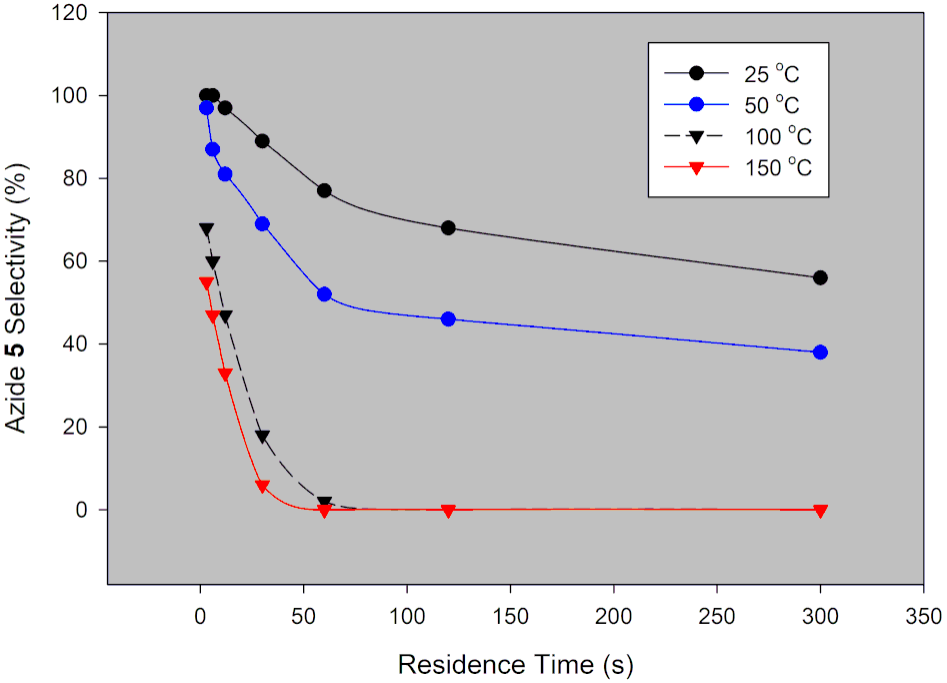
Desired azide **5** selectivity in a continuous-flow system using DPPA.

Using DPPA as the azidating agent, an increase in both temperature and residence time resulted in the increase in mesyl shikimate conversion in microreactors (Figure 8). High temperatures easily gave full mesyl shikimate conversions. Generally, the trends found with the use of DPPA are comparable to NaN_3_. Figure 9 illustrates the reaction selectivity at varying conditions.

**Scheme 4:**
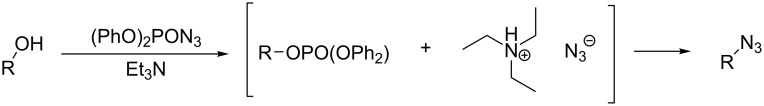
DPPA azidating mechanism in the presence of a base.

Azide **5** selectivity decreases with increase in temperature and residence time (Figure 9). This trend was the same as observed with the use of NaN_3_ as the azidating agent. However, azide **5** selectivity was better with NaN_3_ than DPPA. At 50 °C and 30 s residence time, full conversion was achieved with 95% and 70% azide **5** selectivity using NaN_3_ and DPPA as the azidating agent, respectively. The lower azide **5** selectivity associated with DPPA is a result of the base TEA used. Basic conditions are reportedly detrimental to the azide **5** selectivity [20]. The use of a base in the DPPA procedure was unavoidable as the reaction did not proceed in its absence. This can be understood by considering the DPPA azidating mechanism. The reaction takes place in two discrete steps, the first being phosphate formation followed by azide displacement (Scheme 4) [24].

**Figure 10:**
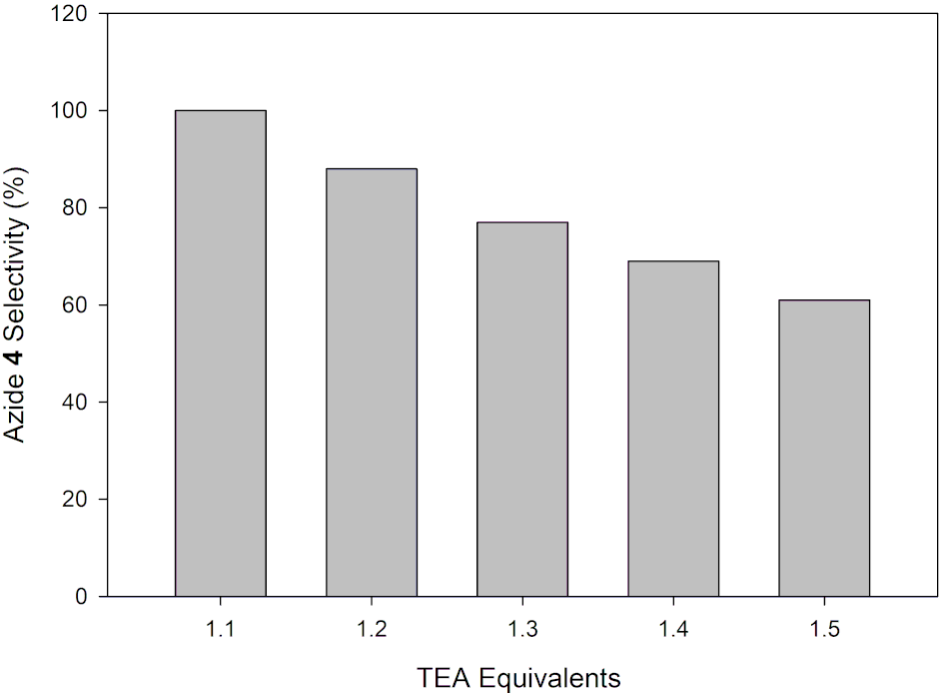
Effect of TEA concentration on the reaction selectivity.

We quenched the reaction within a microreactor using aqueous HCl. Since basic conditions were thought to be detrimental to azide **5** selectivity, investigation of the effect of TEA concentration on the selectivity was reasonable so as to further ascertain this. Therefore, the effect of base (TEA) concentration on azide **5** selectivity was investigated at room temperature and 6 s residence time to ascertain its role in the formation of the unwanted aromatic azide **5a**. Figure 10 illustrates the findings.

The selectivity towards the desired azide **5** significantly decreased with increase in TEA concentration (Figure 10). The aromatization reaction becomes predominant with increasing reaction basicity thus indeed confirming the detrimental effect of basic conditions on the reaction. The use of 1 equiv TEA rather than 1.1 equiv is obviously more logical from the above selectivity study. However, it was accompanied by a 6% conversion loss.

**Figure 11:**
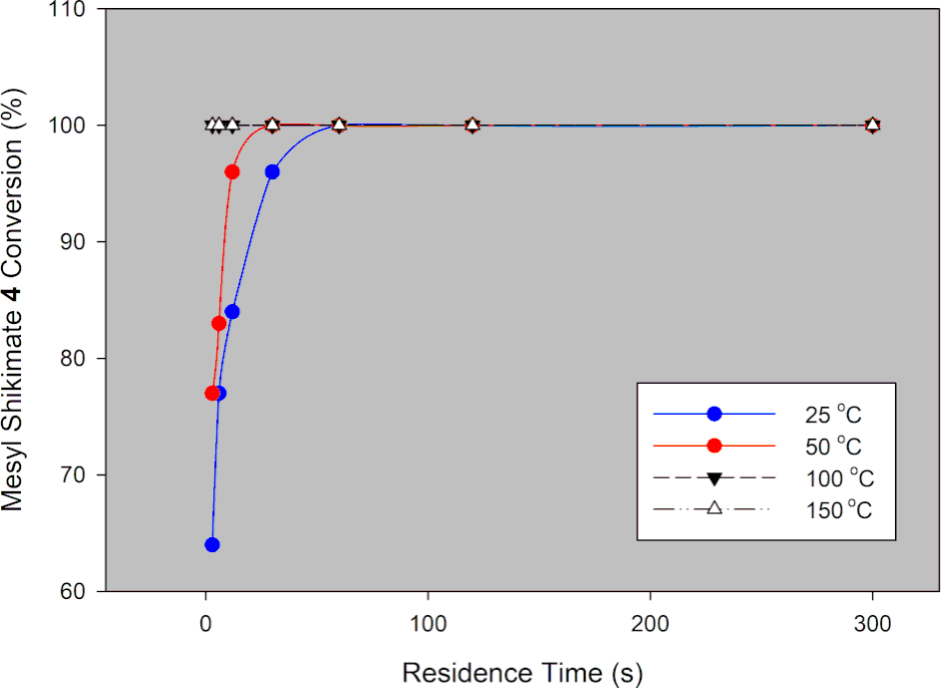
Mesyl shikimate azidation conversion in a continuous-flow system using TMSA.

**Figure 12:**
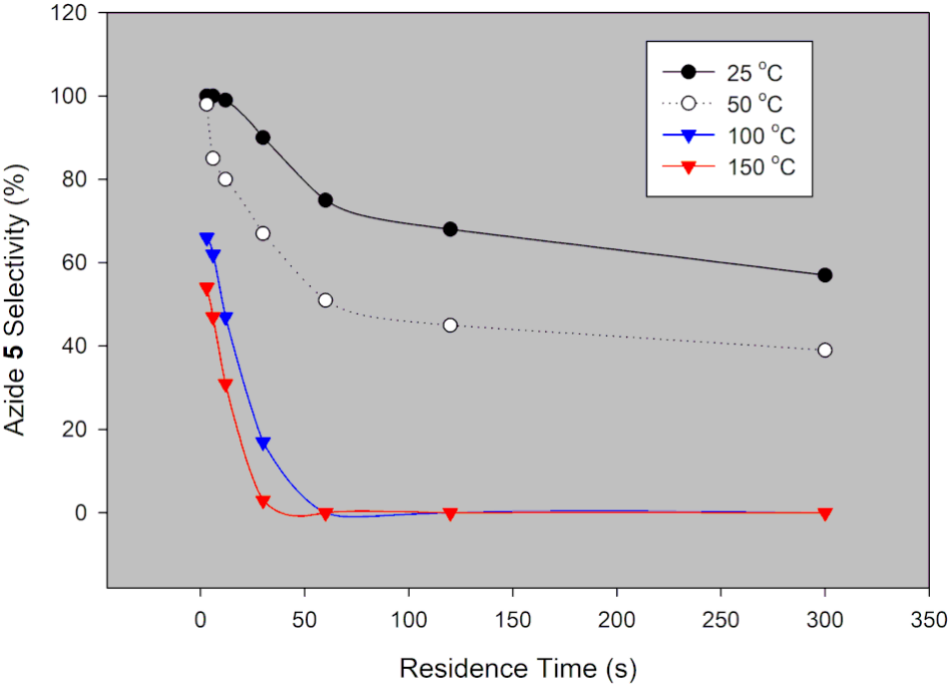
Desired azide **5** selectivity in a continuous-flow system using TMSA.

TMSA was another azidating agent, which was investigated for mesyl shikimate azidation in a continuous-flow system (Figure 7). The reaction conversion and selectivity at varying conditions is presented graphically (Figure 11 and Figure 12).

Just as with the other investigated azidating agents, mesyl shikimate **4** conversion increased with increase in temperature and residence time (Figure 11). The conversions found with TMSA were comparable with both NaN_3_ and DPPA. Mesyl shikimate conversions of 64%, 66% and 71% were obtained at 25 °C and 3 s residence time by using TMSA, DPPA and NaN_3_ as azidating agents, respectively.

The azide **5** selectivity trend using TMSA was similar to DPPA and NaN_3_ (Figures 4, 9 and 12). There is a general decrease in azide **5** selectivity with an increase in temperature and residence time (Figure 12). However, the use of NaN_3_ gave better azide **5** selectivity than TMSA. Azide **5** selectivity of 95% (100% conversion) and 67% (100% conversion) were achieved at 25 °C and 30 s residence time using NaN_3_ and TMSA, respectively. The lower selectivity was because of the basic reaction conditions as TEA was used as a base. The effect of the basic reaction conditions on the selectivity is explained in detail vide supra. Azide **5** selectivity was almost the same when DPPA (69%) and TMSA (67%) was used at 25 °C, 30 s residence time and 100% conversion. It is reasonable since both procedures utilised TEA (1.1 equiv).

We observed that DPPA and TMSA procedures can be used for mesyl shikimate **4** azidation in continuous-flow systems. From a green chemistry point of view, the use of TEA in both procedures was found to be the bottleneck as it lowered azide **5** selectivity. Furthermore, TEA is classified as a non-green reagent, which is not ideal [21–22]. In an effort to resolve this, we investigated the use of TBAA for continuous-flow mesyl shikimate **4** azidation as it does not require a base.

**Figure 13:**
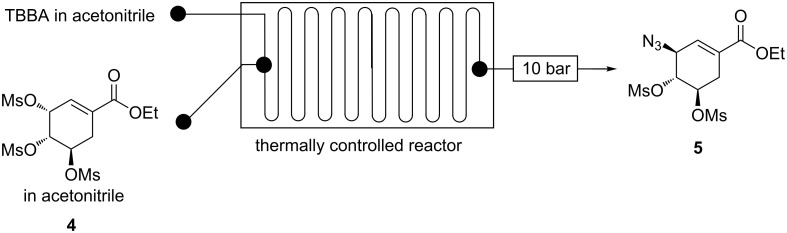
Continuous-flow system for C-3 azidation of mesyl shikimate using TBAA.

**Continuous flow C-3 mesyl shikimate azidation using tetrabutylammonium azide (TBAA).** The use of TBAA as mesyl shikimate **4** azidating agent was investigated in a continuous-flow system (Figure 13).

**Figure 14:**
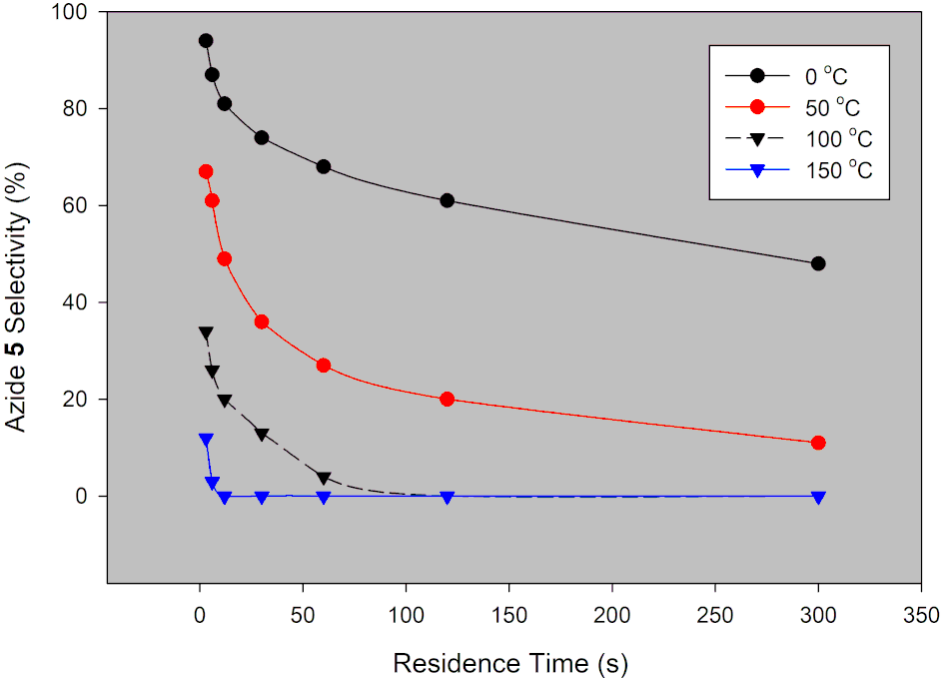
Continuous-flow system for C-3 azidation of mesyl shikimate using TBAA.

Mesyl shikimate **4** (0.1 M) in acetonitrile was treated with TBAA (0.11 M, 1.1 equiv) in acetonitrile in a continuous-flow system (Figure 14). Interestingly, full mesyl shikimate **4** conversion was observed for all investigated reaction conditions, residence time (3–300 s) and temperature (0–150 °C). TBAA proved to be an effective azidating agent for mesyl shikimate **4**. However, there was a variation in azide **5** selectivity under the investigated conditions. The findings on reaction selectivity are presented graphically (Figure 14).

Generally, there is the same azide **5** selectivity trend as the others. Azide **5** selectivity decreased with increase in residence time and temperature (Figure 14). Despite initially promising to be the best azidating agent, this procedure generally gave poor selectivities towards our desired azide **5** compared to the others azidating agents under the same reaction conditions as a result of the basicity of TBAA, making it less green [12]. The optimum conditions are 3 s and 0 °C affording full conversion and 94% azide **5** selectivity. Using TBBA, it was important to quickly analyse the sample soon after collection as the reaction continues in the collection vial at room temperature. Azide **5** selectivity was reduced with time in the vial. Therefore, the development of a suitable quenching procedure may be helpful. The TBAA procedure seems to be a promising NaN_3_ procedure replacement when considering the aforementioned anhydrous telescoping due to its superior azidating power.

Various safe and selective procedures for the synthesis of azide **5** from mesyl shikimate **4** in continuous-flow systems were successfully developed. NaN_3_ (aq) is the best azidating agent for mesyl shikimate **4** towards the desired azide **5**. The optimum conditions are 1.1 equivalents of NaN_3_, 50 °C and 12 s affording full conversion (HPLC) towards the desired azide **5** in 91% isolated yield. Unlike all literature procedures [9,19–20], side product **5a** was not detected using our procedure at 50 °C and 12 s residence time. The use of green solvents, excellent selectivities, safe handling of potentially explosive intermediates rendered the overall process green. The reported batch procedures afforded azide **5** in 91–93% yield over an average of 3 h [9,19–20] which evidently makes our continuous flow procedure more superior. Our procedure could have benefited from the ‘fast and hot’ strategy, which is exclusive to flow chemistry technology. In this strategy, reagents are passed through a heated zone under high temperature at very fast flow rates allowing for rapid reaction completion and is out of the heated zone before significant byproduct is formed [25]. NaN_3_ is the cheapest and greenest azidating reagent of all the developed procedures, however, the drawback is that it is not possible to integrate with the next step, but if this is the case then that strategy will have to be implemented. Basic reaction conditions and high temperatures promote the unwanted aromatisation reaction. There was no aromatisation detected when pure azide **5** was heated. This means that basic conditions promoted OMs elimination and subsequent aromatisation of azide **5** at high temperatures. Our azidating procedures described herein are superior to the reported long batch procedures (2–4 h) [9,19–20].

**Scheme 5:**
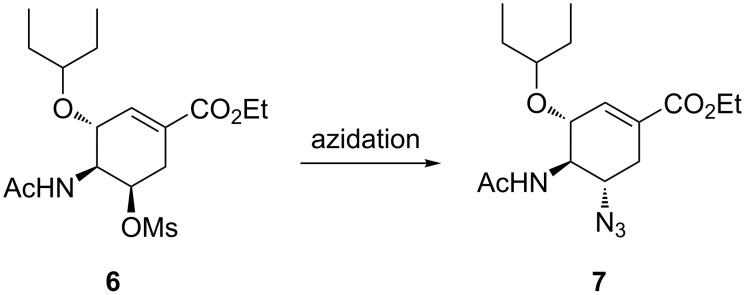
C-5 azidation of acetamide **6** in our proposed route.

**Continuous-flow synthesis of ethyl (3*R*,4*S*,5*S*)-5-azido-4-acetylamino-3-(1-ethylpropyloxy)cyclohex-1-enecarboxylate (7):** The azidation of acetamide **6** is another azidation step in our proposed Tamiflu synthesis route. Acetamide **6** is treated with a suitable azidating agent to afford azide **7**. The C-5 OMs group on acetamide **6** undergoes nucleophilic replacement by the N_3_ group (Scheme 5).

Karpf and Trussardi [9] reported acetamide **6** treatment with NaN_3_ (2 equiv) in a solvent mixture of DMSO and EtOH at 90 °C for 20 h affording azide **6** in 66% yield in batch. Nie et al. [19] demonstrated batch azidation of acetamide **6** with NaN_3_ (4 equiv) in EtOH/H_2_O (5:1) under reflux for 8 h to afford azide **7** in 88% yield. Kalashnikov and co-workers [20] reported the treatment of acetamide **6** in batch with NaN_3_ (3 equiv) by refluxing the reaction in 78% aqueous ethanol for 15 h to afford 95% of azide **7**. Nie and Shi [23] also reported a 3 h acetamide **6** azidating batch procedure using NaN_3_ (4 equiv) in DMF/H_2_O (5:1) affording 84% azide **7**.

Herein, we present acetamide **6** azidation using various azidating agents in a continuous-flow system.

**Figure 15:**
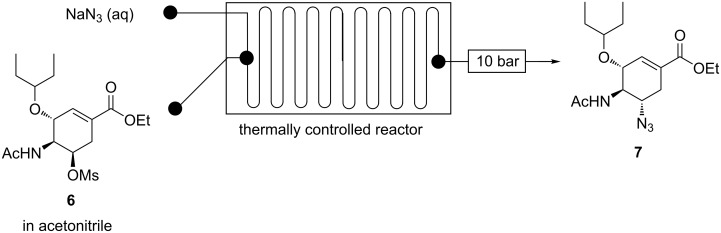
Continuous flow system for C-5 azidation of acetamide **6** using NaN_3_.

**Continuous flow acetamide 6 C-5 azidation using NaN_3._** Acetamide **6** was treated with NaN_3_ in a Chemtrix continuous-flow system affording azide **7** (Figure 15).

**Figure 16:**
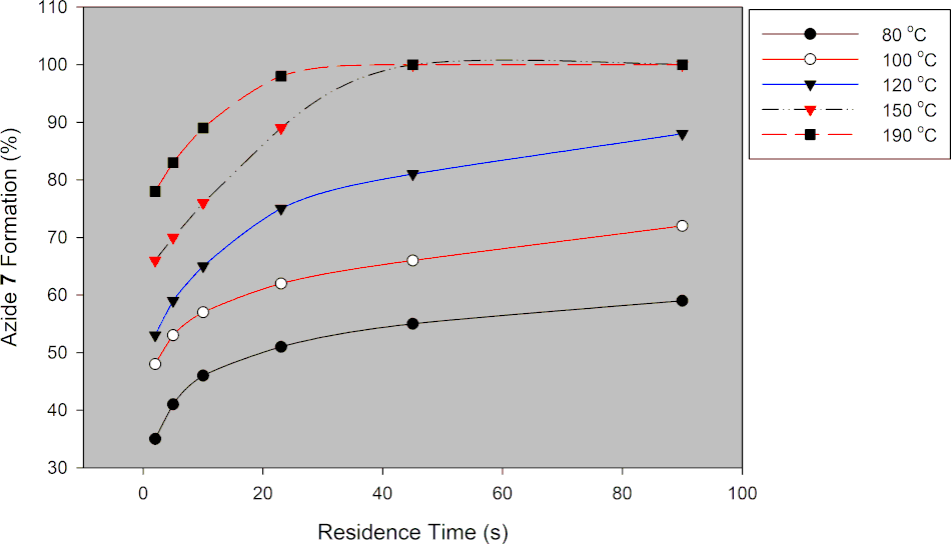
Continuous-flow C-5 azidation of acetamide **6** using NaN_3_.

Guided by batch literature [9,19–20,23], preliminary experiments in flow were done using acetamide **6** (0.1 M) in DMF and aqueous NaN_3_ (0.3 M, 3 equiv) in a 19.5 µL glass microreactor at 100 °C for 90 s affording azide **7** (63%). We achieved 59% of azide **7** when we replaced the hazardous DMF with acetonitrile. The amount of azide **7** produced decreased (44%) when less NaN_3_ (2 equiv) was used. The reaction was further optimised using acetonitrile as acetamide **6** (0.1 M) solvent and aqueous NaN_3_ (0.1 M, 3 equiv) in the Chemtrix 19.5 µL glass reactor (Figure 15). The findings are graphically presented in Figure 16.

As expected azide **7** formation is a function of temperature and residence time (Figure 16). The conversion of acetamide **6** to azide **7** increased with increased temperature. Conversion towards azide **7** was 55% and 100% at 80 °C and 190 °C at 45 s residence time, respectively. At 80 °C, azide **7** was formed in 35% and 59% conversion, respectively (Figure 16). The hazardous DMF was successfully substituted with greener acetonitrile. The optimum conditions were found to be 190 °C and 45 s residence time to afford azide **7** in full conversion (HPLC) and 89% isolated yield. In batch, good yields (66–95%) were attained at reaction times between 3 h and 15 h at temperatures around 90 °C [9,19–20,23]. Our flow procedure was therefore more efficient than all the reported batch procedures. Continuous flow allowed for higher reaction temperatures than batch, which resulted in faster reactions.

**Figure 17:**
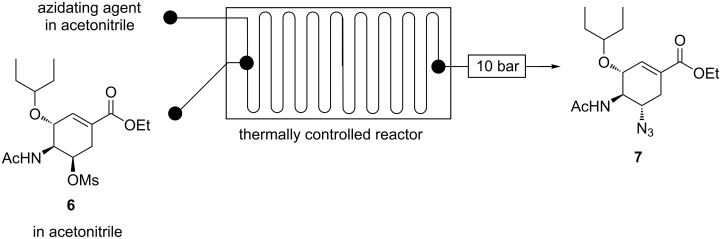
Continuous flow C-5 azidation of acetamide **6** using various azidating agents.

**Continuous flow acetamide 6 C-5 azidation using various azidating agents.** The use of other azidating agents other than NaN_3_ was also investigated in a continuous-flow system (Figure 17).

**Figure 18:**
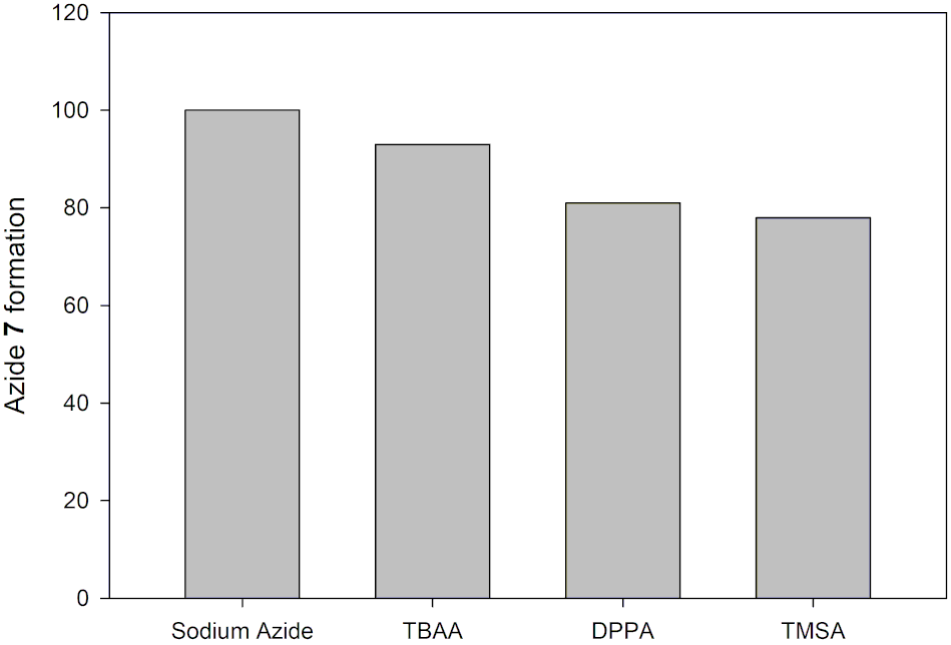
Continuous flow synthesis of azide **7** from acetamide **6** using various azidating agents.

Optimum conditions found for NaN_3_ (1 M, 3 equiv, 190 °C, and 45 s) were used to investigate the use of DPPA, TMSA and TBAA as azidating agents for acetamide **6** (0.03 M) in a 19.5 µL glass microreactor (Figure 17). These azidating agents were dissolved in acetonitrile, not in water as with NaN_3_. Reagents flow rates were used to achieve the required reagents equivalents in flow. Experimental details for this study are outlined in the chapter Experimental. The findings of this study are graphically presented in Figure 18.

We successfully azidated acetamide **6** affording azide **7** at varying conversions (%) using azidating agents (TBAA, DPPA and TMSA) other than NaN_3._ NaN_3_ proved to be the best azidating agent (Figure 18). It is evident that the application of ionic bonded azides (NaN_3_ and TBAA) gave almost similar conversion, 100% and 93%, respectively. Whilst the use of covalently bonded azides (DPPA and TMSA) resulted in lower conversions, 84% and 81%, respectively (Figure 18). NaN_3_ is the azidating agent of our choice on efficiency, affordability and availability viewpoint. TBAA can be used as azidating agent instead of NaN_3_ when anhydrous conditions are required which are useful in multistep syntheses.

Azide **7** was successfully synthesised from acetamide **6** in a continuous-flow system using NaN_3_ as the azidating agent. Optimum conditions for this reaction were found to be NaN_3_ (3 equiv), 190 °C and 45 s residence time affording full conversion (HPLC) towards azide **7** in 89% isolated yield. The use of NaN_3_ (2 equiv) was accompanied by a 15% decrease in conversion. However, full conversion (NaN_3_ (2 equiv)) was achieved at a longer residence time (75 s) than using 3 equiv of NaN_3_ (45 s). Our continuous-flow azidation procedure for acetamide **6** proved to be more efficient than all the literature procedures [9,19–20,23]. Furthermore, it is a green process as full conversion of potentially explosive azide **7** was achieved by using green solvents water and acetonitrile. The reaction temperatures for C-3 azidation (mesyl shikimate **4** azidation, 25 °C, vide supra) differed dramatically with the C-5 azidation (acetamide azidation, 190 °C). This is because the nucleophilic (–N_3_) attack on C-3 (allylic position) is easier than that on C-5 (non-allylic position). Generally, we developed a safe and attractive continuous-flow procedure for the synthesis of azide **7** from acetamide **6**.

## Conclusion

Continuous-flow technology allowed for safe handling of the potentially explosive azide chemistry is involved in our proposed Tamiflu synthesis. Highly efficient, green continuous-flow azide chemistry processes were successfully developed for this study. This useful technique can be utilised for good synthetic approaches towards Tamiflu, which were previously ruled out for large scale synthesis in batch systems on the basis of safety concerns poised by the use of the potentially explosive azide chemistry and other hazardous chemistry. Therefore, problems inherent in scale-up are effectively eliminated or reduced, making microreactor technology a viable tool in the synthesis of Tamiflu. The azide intermediates were safely synthesised in full conversions and >89% isolated yields.

## Experimental

Chemicals were supplied by Sigma-Aldrich, Merck and Industrial Analytical and used as received. Anhydrous solvents were supplied by Sigma-Aldrich and maintained by drying over appropriate drying agents. Nuclear magnetic resonance (NMR) spectra were recorded at room temperature as solutions in deuterated chloroform (CDCl_3_). A Bruker Avance-400 spectrometer (400 MHz) was used to record the spectra and the chemical shifts are reported in parts per million (ppm) with coupling constants in Hertz (Hz). Infrared spectra were recorded from 4000 to 500 cm^−1^ using a Bruker spectrometer and peaks (ν_max_) reported in wavenumbers (cm^−1^). Melting points of all compounds were determined using a Stuart^®^ Melting Point Apparatus SMP30 and Agilent Zorbax C_18_, 10 μm, 4.6 mm × 250 mm column. Continuous-flow reactions were performed on a Labtrix^®^ Start system and a Uniquis FlowSyn system. Reactions were monitored by Agilent 1200 high-performance liquid chromatography (HPLC) fitted with a UV–vis detector. HPLC analysis was performed on Agilent Zorbax C_18_-column (250 mm × 4.6 mm i.d, 5 µm) ambient temperature using an isocratic system. Analysis of collected samples was done using HPLC method (mobile phase consisted of 70% acetonitrile and 30% water. The sample injection volume was 5 µL, eluted at a flow rate of 1.5 mL/min and detected at 213 nm with a run time of 15 min).

**Continuous-flow synthesis of ethyl (3*S*,4*R*,5*R*)-3-azido-4,5-bis(methanesulfonyloxy)cycohex-1-enecarboxylate (5)**. All the mesyl shikimate **4** azidation investigations were done in a continuous-flow system fitted with a 19.5 µL glass reactor for optimisation of the azidation of the OMs group at the allylic C-3 position of mesyl shikimate **4** in the presence of various azidating agents. Sodium azide (NaN_3_), diphenylphosphoryl azide (DPPA), trimethylsilyl azide (TMSA) and tetrabutylammonium azide (TBAA) were the various azidating agents investigated in this system. Two syringe pumps were used to pump reagents from two 10 mL SGE Luer lock gas tight glass syringes into the thermally controlled microreactor system which was fitted with a 10 bar back pressure regulator. Mesyl shikimate was dissolved in acetonitrile (0.1 M) and azidating agent in appropriate solvent (0.11 M, 1.1 equiv) and pumped into the flow system separately. The reaction was quenched within the flow reactor using aqueous HCl (0.11 M, 1.1 equiv) when necessary. Samples were collected and analysed using HPLC resulting in 4.84 min retention time for azide **5**. For characterisation, **an** appropriate amount of toluene and water was added to the reaction mixture after acetonitrile was driven off in vacuo at room temperature. The organic layer was successively washed with water and brine. The organic phase was dried over anhydrous Mg_2_SO_4_ and concentrated in vacuo at room temperature. The crude product was purified by silica column chromatography using a 1:2 mixture of EtOAc and hexane to furnish azide compound **5** as a colourless oil. FTIR (cm^−1^) ν: 2984, 2941, 2105, 1711, 1660, 1350, 1245, 1171, 1011, 823; ^1^H NMR (400 MHz, CDCl_3_) δ 1.25 (t, *J* = 7.0 Hz, 3H), 2.56–2.66 (m, 1H), 3.08 (s, 3H), 3.12 (dd, *J* = 6.2 Hz, 1H), 3.16 (s, 3H), 4.18 (q, *J* = 7.0 Hz, 2H), 4.23–4.31 (m, 1H), 4.64–4.74 (m, 1H), 4.77–4.88 (m, 1H), 6.69 (s, 1H) ppm; ^13^C NMR (100 MHz, CDCl_3_) δ 14.1, 31.1, 39.0, 39.4, 61.1, 61.9, 73.8, 79.1, 130.3, 131.9, 164.17 ppm.

**Continuous flow systhesis of ethyl (3*R,*4*S,*5*S*)-5-azido-4-acetylamino-3-(1-ethylpropyloxy)cyclohex-1-enecarboxylate (7).** The continuous-flow system fitted with a 19.5 µL glass reactor was used to optimise the C-5 azidation of acetamide **6**. Acetamide **6** (0.1 M) in acetonitrile and azidating agent in appropriate solvent (0.3 M, 3 equiv) were pumped separately using two syringe pumps from two 10 mL SGE Luer lock gas tight glass syringes into the thermally controlled microreactor system which was fitted with a 10 bar back pressure regulator. NaN_3_, DPPA, TMSA and TBAA were the various azidating agents investigated. Samples were collected and analysed using HPLC resulting in 4.84 min retention time for azide **5**. For characterisation, the reaction mixture was collected and ethyl acetate and water were added. The organic layer was separated, washed with brine and dried over anhydrous Mg_2_SO_4_. The residue was concentrated in vacuo to yield crude product **7** as a semi-crystalline oil. It was purified by silica column chromatography and the fractions were concentrated in vacuo to afford pure compound **7** as white crystals. Solid: mp 136.9–138.2 °C (Lit. value [9] mp. 136.6–137.7 °C); FTIR (cm^−1^) ν: 3266, 2971, 2099, 1713, 1658, 1558, 1249, 1075; ^1^H NMR (400 MHz, CDCl_3_) δ 0.91 (m, 6H), 1.31 (t, *J* = 7.1 Hz, 3H), 1.42–1.63 (m, 4H), 2.10 (s, 3H), 2.18–2.33 (m, 1H), 2.88 (dd, *J* = 5.5, 17.6 Hz, 1H), 3.29–3.37 (m, 1H), 3.41–3.52 (m, 1H), 4.23 (q, *J* = 6.97 Hz, 3H), 4.49–4.63 (m, 1H), 6.45–6.70 (m, 1H), 6.80 (s, 1H) ppm; ^13^C NMR (101 MHz, CDCl_3_) δ 9.3, 9.5, 14.2, 23.2, 25.6, 26.3, 30.7, 57.3, 57.9, 61.1, 73.6, 77.2, 82.1, 128.1, 137.9, 165.7, 171.76 ppm.

## References

[1] Magano J (2011). Tetrahedron.

[2] Magano J (2009). Chem Rev.

[3] Ogasawara S, Hayashi Y (2017). Synthesis.

[4] Sagandira C R, Watts P (2018). J Flow Chem.

[5] Abrecht S, Federspiel M C, Estermann H, Fischer R, Karpf M, Mair H-J, Oberhauser T, Rimmler G, Trussardi R, Zutter U (2007). Chimia.

[6] Karpf M, Trussardi R (2001). J Org Chem.

[7] Weng J, Li Y-B, Wang R-B, Li F-Q, Liu C, Chan A S C, Lu G (2010). J Org Chem.

[8] Farina V, Brown J D (2006). Angew Chem, Int Ed.

[9] Karpf M, Trussardi R (2009). Angew Chem, Int Ed.

[10] Oshitari L, Mandai T (2009). Synlett.

[11] Nie L-D, Wang F-F, Ding W, Shi X-X, Lu X (2013). Tetrahedron: Asymmetry.

[12] Bräse S (2010). Organic Azides: Syntheses and Applications.

[13] Ishikawa H, Suzuki T, Orita H, Uchimaru T, Hayashi Y (2010). Chem – Eur J.

[14] Ishikawa H, Bondzic B P, Hayashi Y (2011). Eur J Org Chem.

[15] Movsisyan M, Delbeke E I P, Berton J K E T, Battilocchio C, Ley S V, Stevens C V (2016). Chem Soc Rev.

[16] Ehrfeld W, Hessel V, Löwe H (2000). Microreactors: New Technology for Modern Chemistry.

[17] Rogers L, Jensen K F (2019). Green Chem.

[18] Sagandira C R, Watts P (2019). J Flow Chem.

[19] Nie L-D, Shi X-X, Ko K H, Lu W-D (2009). J Org Chem.

[20] Kalashnikov A I, Sysolyatin S V, Sakovich G V, Sonina E G, Shchurova I A (2013). Russ Chem Bull.

[21] Prat D, Wells A, Hayler J, Sneddon H, McElroy C R, Abou-Shehada S, Dunn P J (2016). Green Chem.

[22] Byrne F P, Jin S, Paggiola G, Petchey T H M, Clark J H, Farmer T J, Hunt A J, McElroy C R, Sherwood J (2016). Sustainable Chem Processes.

[23] Nie L-D, Shi X-X (2009). Tetrahedron: Asymmetry.

[24] Thompson A S, Humphrey G R, DeMarco A M, Mathre D J, Grabowski E J J (1993). J Org Chem.

[25] Gernaey K V, Cervera-Padrell A E, Woodley J M (2012). Future Med Chem.

